# Development of an Android-Based Self-Report Assessment for Elderly Driving Risk (SAFE-DR) App: Mixed Methods Study

**DOI:** 10.2196/25310

**Published:** 2021-06-17

**Authors:** Ho Sung Hwang, Seong-Youl Choi

**Affiliations:** 1 Department of Occupational Therapy Wonkwang University Gwangju Medical Center Gwangju Republic of Korea; 2 Department of Occupational Therapy Gwangju Women’s University Gwangju Republic of Korea

**Keywords:** Android driving app, driving safety, reliability, self-assessment, validity, mHealth, driving

## Abstract

**Background:**

Self-report assessments for elderly drivers are used in various countries for accessible, widespread self-monitoring of driving ability in the elderly population. Likewise, in South Korea, a paper-based Self-Report Assessment for Elderly Driving Risk (SAFE-DR) has been developed. Here, we implemented the SAFE-DR through an Android app, which provides the advantages of accessibility, convenience, and provision of diverse information, and verified its reliability and validity.

**Objective:**

This study tested the validity and reliability of a mobile app-based version of a self-report assessment for elderly persons contextualized to the South Korean culture and compared it with a paper-based test.

**Methods:**

In this mixed methods study, we recruited and interviewed 567 elderly drivers (aged 65 years and older) between August 2018 and May 2019. For participants who provided consent, the app-based test was repeated after 2 weeks and an additional paper-based test (Driver 65 Plus test) was administered. Using the collected data, we analyzed the reliability and validity of the app-based SAFE-DR. The internal consistency of provisional items in each subdomain of the SAFE-DR and the test-retest stability were analyzed to examine reliability. Exploratory factor analysis was performed to examine the validity of the subdomain configuration. To verify the appropriateness of using an app-based test for older drivers possibly unfamiliar with mobile technology, the correlation between the results of the SAFE-DR app and the paper-based offline test was also analyzed.

**Results:**

In the reliability analysis, Cronbach α for all items was 0.975 and the correlation of each item with the overall score ranged from *r*=0.520 to *r*=0.823; 4 items with low correlations were removed from each of the subdomains. In the retest after 2 weeks, the mean correlation coefficient across all items was *r*=0.951, showing very high reliability. Exploratory factor analysis on 40 of the 44 items established 5 subdomains: on-road (8 items), coping (16 items), cognitive functions (5 items), general conditions (8 items), and medical health (3 items). A very strong negative correlation of –0.864 was observed between the total score for the app-based SAFE-DR and the paper-based Driver 65 Plus with decorrelation scales. The app-based test was found to be reliable.

**Conclusions:**

In this study, we developed an app-based self-report assessment tool for elderly drivers and tested its reliability and validity. This app can help elderly individuals easily assess their own driving skills. Therefore, this assessment can be used to educate drivers and for preventive screening for elderly drivers who want to renew their driver’s licenses in South Korea. In addition, the app can contribute to safe driving among elderly drivers.

## Introduction

Driving increases the autonomy of elderly individuals, which helps them to participate in activities in the local community and at home [1]. Moreover, elderly drivers who have higher independence and community participation show higher life satisfaction [2]; however, aging is accompanied by factors that impede safe driving, such as perceptual, motor, and cognitive impairments [3,4]. Due to health decline, elderly drivers have much higher accident rates than all other age groups and are more likely to suffer severe injury and death in the event of an accident [5,6]. Therefore, it is becoming increasingly important to develop ways of testing their health status and assessing their suitability for driving [7].

Elderly drivers have the self-control to reflect on their driving ability and prevent driving risk themselves [8]. They generally tend to avoid dangerous driving situations, such as long-duration driving, high-speed driving, and uncomfortable driving situations such as driving at night or in the rain [9]. Using these characteristics of elderly drivers, continuing research is being conducted to develop various self-report assessments and test their correlation with driving risks [10].

Self-report assessment plays an important role in elderly drivers’ decision-making on the continuation of driving and also presents the following advantages: individuals can test their status using a relatively simple method, the assessment can be distributed to a broad population online, and it provides rapid feedback [11]. Existing self-report assessments for elderly drivers were previously only available as paper-based tests; they are now available on CD-ROM [12] and via websites [13]. As such, self-assessment services for elderly drivers are shifting away from the conventional method of paper-based testing to a more efficient way that fits social reality, and the new service modalities should be able to provide older drivers with easier access and faster feedback.

Recently, numerous mobile-based health care apps have been developed to aid health care across a wide population [14]. Although the elderly population may have restricted access to mobile-based services, this is outweighed by various benefits, such as diverse access to information, convenience, and reduced social isolation [15]. Mobile-based services for self-report assessments for elderly drivers have not been attempted so far because elderly persons are not familiar with the mobile environment. Nevertheless, according to the mobile technology acceptance model for the elderly aged 65 years and older, they are strongly influenced by surrounding reference social groups (children and relatives) and do not show major differences from other age groups in motivation to access mobile devices [16]. In other words, even though elderly drivers may experience some difficulties performing mobile-based self-report assessments, it is important to investigate the usability of new methods that provide easier access and feedback.

In the United States, Austria, and most European countries, screening policies based on driving self-assessments are widely used for driver’s license renewals for the elderly population; assessments include the Driving Decisions Workbook, Older Driver’s Self-Assessment Questionnaire, Driving Safely While Aging Gracefully, Safe Driving Behavior Measure, and Driver 65 Plus, among others [17-20]. South Korea also has a traffic safety policy that shortens the length of license renewal and provides professional evaluations for elderly drivers [21]. Within these policies, a means of rapidly screening the driving risk for a wide range of elderly people is needed.

Patterns of driving behavior vary depending on sociocultural characteristics, and elderly drivers’ driving abilities are strongly influenced by perceptual ability and culture [22]. Therefore, it is necessary to thoroughly examine the reliability and validity of self-report assessments in which elderly drivers examine their own status within the corresponding sociocultural environment. To this end, self-report assessments have been developed to suit the characteristics of various countries, such as the United States, Canada, and Australia [11,23]. Likewise, the Self-Report Assessment For Elderly Driving Risk (SAFE-DR) was developed as a self-report assessment for elderly drivers suited to the cultural characteristics of South Korea (Multimedia Appendix 1) [24].

With the above evidence, the purpose of this study was to develop and test a mobile version of a self-report assessment app for elderly persons contextualized to the South Korean culture. We then tested its validity and reliability by comparing its results to that of an established, validated, paper-based test.

## Methods

### Study Design

This was a mixed methods study that included a qualitative exploratory method of a focus group to develop a mobile-based version of the SAFE-DR and a cross-sectional survey conducted among elderly South Korean drivers to assess the validity and reliability of a self-administered Android-based mobile app in assessing their driving risk. Before starting the study, the entire protocol was approved by the Kwangju Women’s University Institutional Review Board (No. 1041485-201709-HR-001-29). All participants received and understood an oral and written explanation of the entire study and provided written informed consent to participate.

### Procedure

This study constituted three phases: (1) development and implementation of the SAFE-DR app, (2) testing of the reliability and validity of the SAFE-DR app, and (3) analysis of the correlation between the SAFE-DR app and a paper-based self-report assessment for elderly drivers.

### Phase One: Implementing the SAFE-DR App

#### App Configuration

To implement the SAFE-DR as an Android app, we used the qualitative exploratory method of focus group interactions [25]. The focus group consisted of 2 app developers and 3 professors specializing in elderly driving rehabilitation. The investigators in this study directed communications within the focus group; the professors specializing in elderly driving rehabilitation composed app scenarios, and the 2 app developers used the Android Studio to produce a pilot file based on these scenarios. App scenarios consisted of using functional and timing point of views to describe or visualize interactions between users and mobile systems, and app developers anticipated use episodes before coding into computer languages. Elderly driving experts discussed scenario visualization, content application on a page, score expression as an evaluative result, and explanations to users about items with low scores. The scenario, including the contents discussed and the message sequence chart, was written using the Excel 2016 program (Microsoft Corp) for each app page. Based on this scenario, the app development specialists used a programming language (Java) to construct the app. Next, the whole group participated in a process of qualitative communication to finalize the function and design of the app from the users’ perspective. The app was designed in 4 sections: explanation of evaluation, guidance, and consent to use; data entry of personal and driving characteristics; performance of test through items of each subdomain; and presentation of test results. The test can be completed in about 20 minutes; the app analyzes the subject’s response to each item (based on a 3-point scale) to provide information on the user’s driving ability. It further cautions the driver to be careful or see an expert for a driving ability test. Finally, an app administration website was created for data management.

#### SAFE-DR

The SAFE-DR was developed by SYC to reflect the characteristics of elderly drivers in South Korea [24]. Via a Delphi survey, a 28-person panel selected 44 assessment items and, after analysis of offline reliability and validity, 38 items were included in the final instrument, with subdomains of on-road, coping, and health. Each item was measured on a 3-point Likert scale of agree, disagree, and strongly disagree. The reliability coefficients for all items (on-road, coping, and health subdomains) were 0.906, 0.921, and 0.913, respectively. The instrument demonstrated content, construct, and predictive validity. In the implementation of the app version of the test in this study, we used the 44 items that showed content validity in the paper-based SAFE-DR, collected data from elderly drivers, and assessed the test items. The app provides information to elderly drivers on any reduction in driving abilities and whether they should discuss their abilities with a driving expert. Based on this information, elderly drivers will be able to make decisions as to whether they should seek expert evaluation or exercise caution in situations to practice safe driving.

### Phase Two: Testing the Reliability and Validity of the SAFE-DR App

#### Testing the Reliability and Validity

Construct validity was analyzed to ensure that the hypotheses for the subdomain configuration of SAFE-DR developed offline were consistent with the actual app-based assessment. The reliability of the configuration items for each subarea was analyzed to select items that were inconsistent. To analyze test-retest reliability, the SAFE-DR app was readministered 2 weeks later to the 81 participants who consented to the retest.

#### Participants and Data Collection

Data were collected between August 2018 and May 2019 through the SAFE-DR app made by the focus group and through one-to-one interviews with elderly drivers. Table 1 shows the general characteristics of the focus group. The focus group developed the app by discussing the topics of organizing app scenarios (function, point of view, interaction, and visualization) and organizing elderly driver assessment content (expression of evaluation content, items, and results).

**Table 1:** General characteristics of the focus group (n=5).

Characteristic			Value
**Sex, n (%)**			
	Male	4 (80)	
	Female	1 (20)	
**Age (years), mean (SD)**			42.60 (9.63)
	35-40, n (%)	3 (60)	
	40-45, n (%)	1 (20)	
	45-50, n (%)	0 (0)	
	50-55, n (%)	0 (0)	
	55-59, n (%)	1 (20)	
**Area of expertise, n (%)**			
	App development	2 (40)	
	Elderly driving rehabilitation	3 (60)	
**Experience with app development or elderly driving rehabilitation (years), n (%)**			
	5-10	1 (20)	
	10-15	3 (60)	
	15-20	0 (0)	
	20-25	1 (20)	

Since it is difficult to specify the population of elderly drivers in South Korea, we used convenience sampling and snowball sampling. After obtaining consent from 3 public health centers and 3 general hospitals in Seoul, Gyeonggi-do, and Gwangju, which have large elderly dynamic populations, we installed testing booths and advertised the study to recruit elderly drivers. In addition, we recruited further participants by advertising by word of mouth through the elderly drivers who had taken the test. All individuals who participated in the study received written and verbal guidance for the entire research process and provided written informed consent to participate in the study.

Participants were enrolled between August 2018 and May 2019, and a total of 567 elderly drivers participated in the study. All participants were interviewed to check general and driving-related characteristics; they completed the SAFE-DR assessment themselves using the developed Android app. In addition, participants who consented to further testing completed an offline self-report assessment using the Driver 65 Plus. Individuals met the inclusion criteria if they were individuals aged 65 years and over who possessed a driving license and had at least 1 year of driving experience and whose cognitive and verbal abilities imposed no restrictions on completing the app-based self-report assessment. Based on the inclusion criteria, of the 567 participants, 51 persons were excluded because they were under 65 years of age or provided incomplete responses to the self-report assessment. The remaining 516 participants were included in the final analysis.

### Phase Three: Analyzing the Correlation Between the SAFE-DR App and a Paper-Based Self-Report Assessment for Elderly Drivers (Driver 65 Plus Test)

#### Correlation Between App and Paper-Based Assessment

The third phase was to test the validity of the assessment results of the elderly drivers, who may not be familiar with mobile apps, by analyzing the correlation between the results of the SAFE-DR app and a paper-based offline test. Among the elderly drivers who used the SAFE-DR app, those who consented to further testing were asked to complete the Korean version of the Driver 65 Plus test, which is one of the most widely used offline self-report assessments for elderly drivers. The correlation between the SAFE-DR app and paper-based Driver 65 Plus results was analyzed to verify consistency between the results of the offline and online tests. In the Driver 65 Plus test, lower scores indicated a more positive result, whereas in the SAFE-DR test, higher scores indicated a more positive result. Therefore, we expected to observe a negative value for the correlation coefficient between the two tests.

#### Driver 65 Plus

This test was developed by the American Automobile Association based on the Safety Research and Education Project of the Teacher’s College of Columbia University [26]. A feature of this test is that the scoring scale for each item is presented differently depending on the content such that elderly drivers cannot predict the question’s intentions. The Korean Driver 65 Plus has shown test-retest reliability of .95 and construct and concurrent validity [27].

### Statistical Analysis

For data analysis, we used PASW Statistics (version 18.0, IBM Corp). Participant characteristics were analyzed using the descriptive statistics of mean, frequency, and percentage. Cronbach α and the correlation among all items were analyzed to examine the internal consistency of provisional items in each subdomain of the SAFE-DR and the stability of a retest after 2 weeks.

Exploratory factor analysis was performed to classify the subdomains of SAFE-DR. For exploratory factor analysis, we used principal component analysis, selecting factors with an initial eigenvalue ≥1.0, and used a varimax rotation. To interpret the results, we checked whether the Kaiser-Meyer-Olkin value in Bartlett test of sphericity was ≥0.8, that the factor loading of each item was ≥0.4, and that the eigenvalue of each factor was ≥1.0. Although a factor loading ≥0.5 for each item is ideal, given that this was a sociological study, we interpreted the results using a criterion of 0.4 [28,29]. Pearson correlation analysis was used to estimate the correlation coefficients for each item in test-retest reliability and to estimate the correlation between the paper-based self-report assessment results and the app-based self-report assessment results.

## Results

### General Characteristics of Participants

Table 2 shows the general characteristics of the elderly drivers who participated in this study. Of the elderly drivers, 79.7% (411/516) were male and 20.3% (105/516) were female, while the mean age was 72.03 years. A total of 45% (232/516) were taking regular medication for hypertension (187/516, 80.6%), diabetes (25/516, 10.8%), hyperlipidemia (11/516, 4.7%), and prostate disorders (6/516, 2.6%); however, 3 drivers (3/516, 1.3%) were also taking antiepileptic medication, which could affect driving. The automobile types most commonly used by the elderly drivers were sport utility vehicles (168/516, 32.6%), midsize sedans (144/516, 27.9%), and compact cars (114/516, 22.1%). The mean driving time of the 516 participants was 22.75 years, the average driving time per week for the 479 currently driving drivers was 4.49 hours, and the average driving cessation period for the 37 participants who were not currently driving was 1.78 years. A total of 4.4% (21/516) had experienced driving accidents during the last 3 months, of which the majority (13/516, 2.5%) experienced a collision due to their own fault.

**Table 2:** General characteristics of the elderly drivers (n=516).

Characteristic					Value
**Sex, n (%)**					
	Male			411 (79.7)	
	Female			105 (20.3)	
**Age (years), mean (SD)**					72.03 (5.13)
	65-69, n (%)			192 (37.2)	
	70-79, n (%)			269 (52.1)	
	≥80, n (%)			55 (10.7)	
**Regular medicine, n (%)**					
	**Yes**			232 (45.0)	
		Hypertension	187 (80.5)		
		Diabetes	25 (10.8)		
		Hyperlipidemia	11 (4.7)		
		Prostate problems	6 (2.6)		
		Antiepileptic	3 (1.3)		
	No			284 (55.0)	
**Automobile type, n (%)**					
	Compact car			114 (22.1)	
	Midsize sedan			144 (27.9)	
	Sport utility vehicle			168 (32.6)	
	Large size sedan			35 (6.8)	
	Business vehicle			3 (0.6)	
	Other			52 (10.1)	
Driving experience (years), mean (SD)					22.75 (8.04)
**Currently driving**					
	**Yes, n (%)**			479 (92.8)	
		Total driving time per week (hours), mean (SD)	4.37 (2.36)		
	**No, n (%)**			37 (7.2)	
		Period (years), mean (SD)	1.78 (1.45)		
**Driving accident in the past 3 months, n (%)**					
	**Yes**			21 (4.1)	
		Individual accident	1 (0.2)		
		Minor accident (by me)	3 (0.6)		
		Minor accident (by others)	2 (0.4)		
		Collisions (by me)	13 (2.5)		
		Collisions (by others)	2 (0.4)		
	No			495 (95.9)	

### Internal Consistency

To analyze the internal consistency of the SAFE-DR app, we analyzed Cronbach α and the correlation of each item with the overall score. For all items, Cronbach α=.975, and the correlation of each item with the overall score ranged from *r*=.520 to *r*=.823, meaning that all items showed a correlation of at least .50 (Table 3).

**Table 3:** Correlation of individual items to the total Self-Report Assessment for Elderly Driving Risk score and test-retest reliability (n=516).

Pre-subarea and item number		Mean (SD)	Item to total correlation	Cronbach α		Total^a^
				When item is deleted	Test-retest^b^ (n=101)	
**On-road**		—^c^	—	—	—	.921
	1	2.44 (.664)	.418	.924	.932	—
	2	2.35 (.721)	.556	.919	.965	—
	3	2.35 (.736)	.709	.913	.963	—
	4	2.09 (.806)	.738	.912	.961	—
	5	2.10 (.823)	.771	.910	.965	—
	6	2.19 (.781)	.804	.909	.933	—
	7	2.40 (.688)	.771	.911	.941	—
	8	2.41 (.672)	.801	.910	.944	—
	9	2.27 (.764)	.642	.916	.947	—
	10	2.27 (.780)	.703	.913	.925	—
	11	2.30 (.729)	.756	.911	.947	—
	12	2.20 (.739)	.413	.925	.936	—
**Coping**		—	—	—	—	.946
	13	2.34 (.679)	.509	.947	.976	—
	14	2.48 (.628)	.614	.945	.951	—
	15	2.26 (.732)	.687	.943	.930	—
	16	2.30 (.732)	.739	.942	.960	—
	17	2.25 (.722)	.762	.942	.940	—
	18	2.38 (.678)	.551	.946	.919	—
	19	2.21 (.762)	.646	.944	.980	—
	20	2.26 (.726)	.756	.942	.966	—
	21	2.34 (.672)	.772	.942	.918	—
	22	2.08 (.793)	.685	.944	.936	—
	23	2.25 (.742)	.799	.941	.961	—
	24	2.38 (.667)	.782	.942	.945	—
	25	2.19 (.784)	.733	.942	.954	—
	26	2.23 (.751)	.792	.941	.957	—
	27	2.21 (.783)	.722	.943	.952	—
	28	2.25 (.758)	.714	.943	.980	—
**Health**		—	—	—	—	.936
	29	2.23 (.775)	.629	.934	.979	—
	30	2.20 (.753)	.714	.931	.970	—
	31	2.23 (.726)	.680	.932	.941	—
	32	2.29 (.718)	.712	.931	.982	—
	33	2.41 (.658)	.715	.931	.974	—
	34	2.04 (.777)	.629	.934	.942	—
	35	2.23 (.730)	.666	.933	.981	—
	36	2.14 (.769)	.691	.932	.948	—
	37	1.99 (.809)	.682	.932	.945	—
	38	2.03 (.795)	.721	.931	.976	—
	39	2.24 (.754)	.750	.930	.952	—
	40	2.22 (.741)	.708	.931	.938	—
	41	2.28 (.739)	.738	.931	.964	—
	42	2.59 (.585)	.551	.935	.974	—
	43	2.54 (.633)	.604	.934	.909	—
	44	2.67 (.520)	.509	.936	.898	—

^a^Total Cronbach α=.975.

^b^Mean of the total test-retest correlations, r=.951.

^c^Not applicable.

For each of the subdomains defined when the SAFE-DR app was constructed, any item that increased Cronbach α when removed was excluded from the assessment; 5 items showed either increased or equal reliability when removed. For items that showed the same reliability, we made decisions regarding deletion based on subdomain composition in the factor analysis. As a result of this process, we decided to remove items 1, 12, 13, and 18.

### Construct Validity

In the exploratory factor analysis, the Kaiser-Meyer-Olkin value was 0.963, which was ≥0.8 and close to 1, and the eigenvalues of each factor were ≥1.0, which was independently sufficient to form subdomains. The result of Bartlett test of sphericity, which indicates the suitability of the total data in the factor model, was statistically significant (*P*<.001, chi-square: 17,265.9; Table 4).

**Table 4:** Exploratory factor analysis for Self-Report Assessment for Elderly Driving Risk subdomains.

Final subarea and item number				Factor 1		Factor 2		Factor 3		Factor 4		Factor 5		Communality summary loading
**On-road**														
	2		–.028		.059		.692		.275		.260		.626	
	3		.200		.153		.719		.240		.229		.690	
	4		.420		.587		.438		.096		.040		.724	
	5		.377		.477		.574		.131		.066		.721	
	6		.392		.378		.652		.149		.054		.747	
	7		.384		.170		.639		.306		.211		.722	
	8		.379		.241		.641		.273		.187		.722	
	9		.331		.426		.510		–.033		.177		.584	
**Coping**														
	10		.560		.237		.426		.230		.145		.624	
	11		.542		.346		.393		.190		.267		.675	
	14		.405		.053		.330		.187		.514		.575	
	15		.536		.222		.396		.146		.158		.540	
	16		.537		.239		.513		.158		.211		.678	
	17		.513		.422		.351		.247		.176		.656	
	19		.527		.388		.105		.082		.250		.509	
	20		.656		.283		.161		.289		.177		.651	
	21		.564		.294		.333		.280		.227		.645	
	22		.465		.604		.158		.125		.083		.629	
	23		.600		.399		.253		.307		.163		.704	
	24		.540		.329		.301		.366		.208		.668	
	25		.662		.307		.141		.283		.172		.662	
	26		.649		.443		.223		.190		.157		.728	
	27		.682		.370		.159		.175		.046		.660	
	28		.531		.271		.342		.401		.090		.642	
**Health**														
	**Cognitive functions**													
		29	.157		.126		.366		.737		.156		.742	
		30	.447		.275		.337		.505		.122		.659	
		31	.265		.251		.139		.731		.166		.714	
		32	.231		.280		.251		.689		.199		.709	
		33	.311		.242		.140		.602		.378		.680	
	**General conditions**													
		34	.220		.659		.165		.141		.193		.567	
		35	.202		.596		.204		.287		.217		.567	
		36	.233		.703		.205		.200		.166		.658	
		37	.212		.799		.135		.181		.098		.744	
		38	.369		.681		.162		.254		.084		.698	
		39	.372		.516		.138		.478		.118		.666	
		40	.358		.476		.221		.370		.165		.569	
		41	.428		.551		.157		.231		.290		.649	
	**Medical health**													
		42	.161		.175		.186		.158		.784		.730	
		43	.158		.284		.174		.187		.735		.712	
		44	.123		.104		.177		.160		.843		.795	
Eigenvalue				20.585		2.124		1.574		1.324		1.033		—^a^
Variance explained (%)				51.462		5.310		3.935		3.309		2.581		—
Cumulative variance (%)				51.462		56.773		60.707		64.017		66.598		—

^a^Not applicable.

### Factor Exploration

Although a factor loading ≥0.5 for each item is ideal, given that this was a sociological study, we derived a solution that satisfies factor loading ≥0.4. Considering the semantic units of individual items, we decided on a factor solution that did not disturb the semantic units of items included in the theoretically chosen subdomains of on-road, coping, and health. Therefore, during factor analysis, the input arrangement of items was used instead of ordering the items by factor component size. As a result, in the final factor matrix, the initial 3 putative subdomains were divided into 5 subdomains. The on-road and coping subdomains maintained their theoretical arrangements, whereas the items included in the theoretical health subdomain were divided into 5 subdomains. Reviewing the semantic content of the grouped items, we defined items 29-33 as the cognitive functions subdomain, items 34-41 as the general conditions subdomain, and items 42-44 as the medical health subdomain (Table 3).

### Stability

To analyze measurement stability using the SAFE-DR app, we retested the app using the same test after 2 weeks. We analyzed the correlation between initial test results and retest results, and the correlation coefficient ranged from *r*=.898 to *r*=.982. The mean correlation coefficient across all items was *r*=.951 (Table 2).

### Correlation Between App-based SAFE-DR and Driver 65 Plus

To investigate whether elderly drivers, who may not be accustomed to a mobile environment, showed similar trends in the results of the SAFE-DR app and that of the paper-based test, we analyzed the correlation between Driver 65 Plus scores and the total SAFE-DR score with the subdomains. The total SAFE-DR score showed a negative correlation of –0.864 with the Driver 65 Plus score; among subdomains, coping showed the strongest correlation (–0.812). A Pearson correlation coefficient ≥0.8 indicates a very strong correlation. Meanwhile, the on-road, cognitive functions, and general conditions subdomains also showed strong correlations of –0.768, –0.758, and –0.767, respectively. The medical health subdomain showed a significant but moderate correlation of –0.456 (Table 5). Therefore, elderly drivers did not show especially different responses in the app-based method from the paper-based method.

**Table 5:** Correlation between Self-Report Assessment for Elderly Driving Risk and Driver 65 Plus (n=81).

Topic	Driver 65 Plus	App-based SAFE-DR^a^					
		On-road	Coping	Cognitive functions	General conditions	Medical health	Total
Driver 65 Plus	1	—^b^	—	—	—	—	—
On-road	–0.768	1	—	—	—	—	—
Coping	–0.812	0.807	1	—	—	—	—
Cognitive functions	–0.758	0.630	0.788	1	—	—	—
General conditions	–0.767	0.732	0.786	0.758	1	—	—
Medical health	–0.456	0.381	0.414	0.339	0.343	1	—
Total	–0.864	0.882	0.963	0.845	0.887	0.479	1

^a^Self-Report Assessment for Elderly Driving Risk.

^b^Not applicable.

### Final App for SAFE-DR

**Figure 1:**
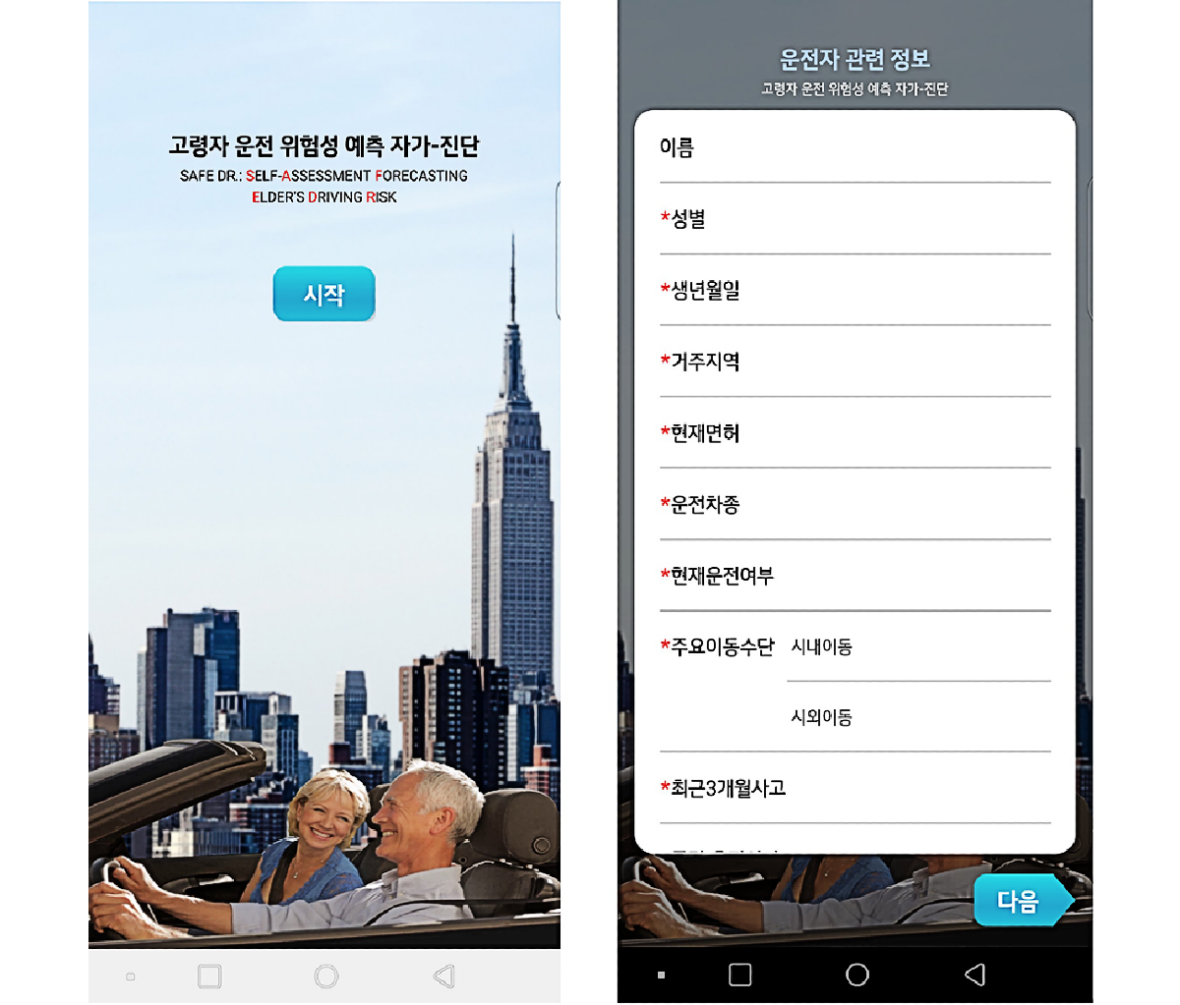
Title and information collection screenshot of the Android app: title page (left) and driver-related information such as gender, date of birth, residence, license, driving status, primary means of transportation, and accident during the past 3 months (right).

**Figure 2:**
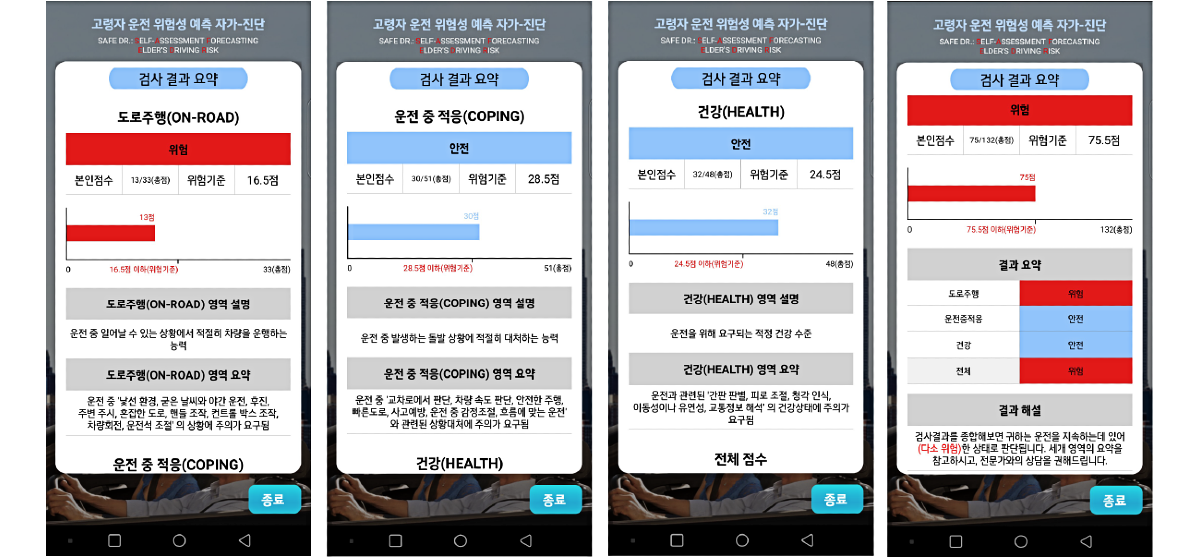
Screenshot of test results in the Android app. Displayed are subarea, total test results (classification of risk or safety in driving, graph of scores against reference points), and information requiring attention.

The final app for SAFE-DR is easily accessible through the Google Play app store. Examples of app screens are shown in Figures 1 and 2.

## Discussion

### Principal Findings

In this study, we developed the SAFE-DR as an app-based self-report assessment for elderly drivers in South Korea. We tested the reliability and validity of the SAFE-DR in a mobile environment and demonstrated that the app-based testing method can be effectively used for elderly drivers.

For the development of the conventional paper-based SAFE-DR, 44 items were tested for content validity by an expert panel in a Delphi survey, and 38 items were selected in the subdomains of on-road, coping, and health. In the app-based SAFE-DR test in this study, 40 out of the 44 items were selected; items 9, 19, and 43 had been excluded from the paper-based test but were not excluded from the app-based test. In the factor analysis in our study, these items showed the lowest communality scores in their respective factors. In exploratory factor analysis, communality is a measure of how well each factor represents a given item [28]. Therefore, these 3 items can be interpreted as showing a relatively low correlation with other items in the same factor in the app-based test as well. Likewise, in the analysis of item reliability, these items tended to show lower reliability. When the paper-based test was developed, a sample of 339 participants was used, whereas, in our study, the sample size was 516 persons; reliability and validity analysis results are more likely to show higher values with a larger sample size [30]. Therefore, although the items showing low reliability and validity were similar between the paper-based and app-based tests, we can cautiously surmise that, due to the larger sample size, the results in our test had higher acceptance. The fact that the results of both the offline and online tests showed similar trends can be considered an important indicator in the development of the app-based test.

This study provides new findings as we implemented the SAFE-DR in an app, collected data, and verified its objectivity. Elderly drivers showed similar trends in the app-based test that were similar to using a paper-based, offline method. The results of the analysis showed a very strong negative correlation between the app-based SAFE-DR and the paper-based Driver 65 Plus; among subdomains of the SAFE-DR, medical health showed a moderate negative correlation and other subdomains showed strong negative correlations. These results suggest that the results of the app-based test have a linear relationship with the results of the paper-based test. Therefore, elderly drivers who may not be familiar with a mobile environment can use the app-based SAFE-DR without any major differences from the paper-based assessment. A study on the use of mobile-based services by elderly populations in various countries indicated that most elderly individuals are already prepared to use mobile-based services; their use reportedly improves independent living among the elderly [31]. Accordingly, it will be necessary to provide various services using a mobile environment such as apps for elderly drivers in Korea as well, including self-report assessments that can provide immediate and faster access to more information.

Some differences were observed between the paper-based test and our study’s app-based test in subdomain composition. We first hypothesized theoretical factors based on the 3 subdomains of the basic paper-based SAFE-DR, and from the performed exploratory factor analysis, 5 factors emerged. This is because, among the putative subdomains, the health subdomain was split into 3 subdomains; these subdomains’ items are related to fatigue during driving, driving-related cognition, visual perception, physical ability, and medical health status, including medication. The fact that health factors were further divided indicates that for the participants in our study the response to driving-related health status differed depending on specific health factors. Items 29-33, which were combined into one factor, were defined as the cognitive functions subdomain, as they shared content relating to memory, cognition, and geographical orientation. Items 34-41 were defined under the broad heading of general conditions, as they shared content on fatigue during driving, visual perception, auditory perception, physical fatigue, and flexibility. Finally, items 42-44 were defined as the medical health subdomain, as they showed content relating to medication, doctor consultations, epilepsy, and seizures.

The heterogeneity of digital literacy in the elderly population’s use of mobile phones prevents older people from accessing smartphones. In a study on attitudes regarding mobile phone use in the daily lives of the elderly, there are individual differences in accessibility to mobile phones. If they refused to use a mobile phone, they felt uncomfortable reading the text on the touch screen. However, there were individuals who learned through personal networks and user manuals and used mobile phones [32]. In this respect, we tried to improve the visibility for older adults in app-based SAFE-DR configurations. Specifically, we increased the font size, reduced the number of characters displayed per screen, and encouraged the use of scroll functions. In a society where single-person mobile phone use has become routine, the elderly, like the populations of other age groups, can no longer avoid the use of smartphones. Therefore, the elderly should continue to try to use mobile devices. In this study, there was a difference between the paper-based evaluation and the app-based evaluation, but it was not a critical issue and it can be modified through feedback from elderly users. The app is currently being distributed through Google Play and is available free of charge. The researcher will continue to maintain this service for elderly drivers and plans to supplement it through feedback from elderly users.

### Comparison With Prior Work

Regarding the subdomain compositions of previous self-report assessments for elderly drivers, the Driving Decisions Workbook developed in the United Kingdom contains on-road, seeing, thinking, getting around, and health subdomains [33]; the Self-Awareness and Feedback for Responsible Driving developed by the US Department of Transportation contains seeing, thinking, and getting around subdomains [13], and the Royal Automobile Club of Queensland Older Drivers’ Self-Assessment Questionnaire developed by the Australian Automobile Association contains driving and health subdomains [34]. Among the subdomains in our study, on-road was included using the same name as previous assessments, and coping refers to the ability to cope with specific situations that can occur while driving on the road, which is consistent with the previous getting around subdomain. For health-related items, the cognitive functions subdomain in our study has similar content to the items in the thinking subdomains in previous assessments. Conversely, the general conditions and medical health subdomains showed differences from those of previous assessments; items in the previous seeing subdomain were partially included in general conditions. Content in the medical health subdomain relating to medication or doctor consultations was previously included in the health subdomain in other assessments; however, since the division of roles between doctors and pharmacists in South Korea, the rate of patients seeking preventive medication guidance or consultations with doctors before disease or an accident has declined [35]. It is thought that these items were classified as a single factor, because unlike in other countries, elderly drivers showed different health status and different patterns in questions related to their medical state. This supports the idea that the app-based SAFE-DR test developed in this study properly reflects the cultural circumstances in South Korea.

The Australasian Model License Assessment Procedure states that the ideal procedure for driver’s license renewal for elderly drivers follows a process of applying for testing through the local community, medical testing, multistage driving ability assessment, and provision of license options [36]. In this testing process, self-report assessments are used to screen the elderly person’s driving ability in the phase of applying for testing through the local community to verify a potential need for specialized tests [37]. Based on this need, various countries use self-assessment tools to monitor the population of elderly drivers [12,13]. Likewise, the SAFE-DR was developed in South Korea as a self-report assessment for elderly drivers suited to the local culture [14], and our study has demonstrated the reliability and validity of a SAFE-DR test provided in an app. Compared to assessments that require meeting with a professional, self-assessment tools allow individuals to test their own abilities, which has the advantage of reducing stress and readily identifying potential problems in a broad population of elderly drivers [23]. In addition, mobile-based services can provide the elderly with various benefits such as access to diverse information, convenience, and reduced social isolation [16]. Therefore, this study’s app-based SAFE-DR test can be used for South Korean elderly drivers both to conveniently test their own driving ability and determine the need for further testing.

Although services provided in a mobile environment have the advantage of easy, repeated access, if the results are not consistent each time the service is used, the user may become confused. Therefore, we analyzed the test-retest reliability when testing was repeated after 2 weeks; most items showed a very high reliability of ≥0.9, demonstrating the test’s ease of access and repeated use.

### Limitations

Our study has some limitations. We were unable to control certain factors during participant recruitment, resulting in a high ratio of males, a low proportion of persons aged ≥80 years, and a low proportion of elderly drivers who were not currently driving. Since we were developing an assessment to be used by South Korean elderly drivers, it was necessary to recruit a better-matched sample of participants and compare the test results according to participants’ characteristics. In addition, because we only provided an app-based service for Android, it will be necessary to expand the test to other platforms to allow use by a larger population.

### Key Findings

Despite these limitations, this study developed, for the first time, the SAFE-DR into an app-based self-report assessment that reflects the cultural characteristics of Korean elderly drivers. While many mobile-based services have recently been offered due to the advantages of easy access and fast information delivery, elderly persons who prefer conventional ways may have difficulty using such services [38]. This study confirmed that the results of both the app-based SAFE-DR evaluations and the paper-based assessments, which are familiar to the elderly, were consistent. Further, this study identified the factors for screening the driving abilities of Korean elderly drivers and tested the reliability for repeated use of self-assessment apps.

This assessment has implications on policies and traffic safety for Korean elderly drivers. Restrictions on the license of elderly drivers are beneficial for their traffic safety and that of the general population; however, these policies should include methods for education and preventive inspections of equipment to help maintain the licenses of elderly drivers as long as possible [39]. The app-based evaluation in this study can be used as means for preventive monitoring and education of the elderly driver population. Therefore, the assessment can help with the education and screening sections of the recent policy for renewing elderly drivers’ licenses in South Korea [21]. Within these policies, this assessment can contribute to the safe continuation of driving by facilitating testing of driving ability and providing relevant information to elderly drivers in South Korea. Finally, future research should focus on expert driving ability assessment and licensing restriction systems for the policy on renewal of elderly Korean’s driver’s licenses.

### Conclusions

In this study, we developed the SAFE-DR into an app-based self-report assessment for elderly drivers and tested its reliability and validity. In South Korea, where the aging population is rapidly increasing, the app can help elderly drivers to easily diagnose their driving skills and protect themselves from accidents while driving. It was designed to be easily used by elderly drivers and to provide essential information related to driving. Therefore, we anticipate that this assessment can contribute to safe continuation of driving by facilitating testing of driving ability and providing relevant information to elderly drivers in South Korea.

## Appendix

Multimedia Appendix 1 Self-Report Assessment for Elderly Driving Risk (SAFE-DR) questionnaire.

## Abbreviations

SAFE-DR: Self-Report Assessment for Elderly Driving Risk
