# Supplementary material for: Development of an Android-Based Self-Report Assessment for Elderly Driving Risk (SAFE-DR) App: Mixed Methods Study
Source: JMIR Mhealth Uhealth. 2021 Jun 17;9(6):e25310. doi: 10.2196/25310 (PMC8277309; doi:10.2196/25310)
Supplement: Multimedia Appendix 1 [file mhealth_v9i6e25310_app1.docx]

**Appendix 1: SAFE-DR Questionnaire**

| **SAFE-DR (Self-Assessment Forecasting Elderly Driving Risk)** | | | | | | | | | | | | | | | | | | | | | | |
| --- | --- | --- | --- | --- | --- | --- | --- | --- | --- | --- | --- | --- | --- | --- | --- | --- | --- | --- | --- | --- | --- | --- |
|  | | | | | | | | | | | | | | | | | | | | | | |
| **Driving-related information** | | | | | | | | | | | | | | | | | | | | | | |
| **Name** | |  | | | | | | **Date of assessment** | |  | **M** | | |  | | **D** | |  | | | **Y** |  |
| **Gender** | |  | | | | | | **Age** | |  | | | | **Years old** | | | | | | | | |
| **Type of driver’s license** | |  | | | | | | **Driving experience** | |  | | | | | **Y** |  | | | | **M** | | |
| **Main mode of transportation** | | **In the city** | | |  | | | **Accident within 3 years prior** | | **Yes** | | | | | **□** | **No** | | | | **□** | | |
|  |  | **Out of the city** | | |  | | | **Cause of the accident** | |  | |  | | | | | | | | | | |
| **Currently driving** | | **Yes** | **□** | **No** | | | **□** | **Regularly taking medicine** | | **Yes** | | | | | **□** | **No** | | | | **□** | | |
| **Number of hours driving per week** | |  | | | |  | | **Medication taken** | |  | | | | | |  | | | | | | |
|  |  |  |  |  |  |  | |  |  |  |  |  |  |  |  |  | | | | | | |
|  | | | | | | | | | | | | | | | | | | | | | | |
| **Score scale** | | **1point = agree.** | | | | | **2points= disagree.** | | **3points= Strongly disagree.** | | | | | | | | | | | | | |
|  | | | | | | | | | | | | | | | | | | | | | | |
| **Item** | **Part 1. On-road** | | | | | | | | | | | | **Score** | | | | | | | | | |
| **1** | You have difficulty adjusting the driver's seat, considering the distance from the steering wheel and the field of view, etc. | | | | | | | | | | | ① | | | | | ② | | ③ | | | |
| **2** | You have difficulty operating control box (e.g. radio, head light, switch) during vehicle. | | | | | | | | | | | ① | | | | | ② | | ③ | | | |
| **3** | You have difficulty driving in certain conditions (e.g., weather conditions, night driving). | | | | | | | | | | | ① | | | | | ② | | ③ | | | |
| **4** | You have difficulty driving in unfamiliar environments (e.g., with use of a map, navigation, signs). | | | | | | | | | | | ① | | | | | ② | | ③ | | | |
| **5** | You have difficulty in reversing the car (e.g. viewing mirror while reversing, going to the right place). | | | | | | | | | | | ① | | | | | ② | | ③ | | | |
| **6** | You have difficulty rotating the steering wheel (e.g., left, right) while driving. | | | | | | | | | | | ① | | | | | ② | | ③ | | | |
| **7** | You have difficulty fixing the steering (e.g. keeping the lane straight or in a turn) wheel during driving. | | | | | | | | | | | ① | | | | | ② | | ③ | | | |
| **8** | You use the rear-view instead of side mirrors, to check traffic and change lanes. | | | | | | | | | | | ① | | | | | ② | | ③ | | | |
|  | | | | | | | | | | | | | | | | | | | | | | |
| **Subtotal** | | | | | | | | | | | | |  | | | | | **/ 24 points** | | | | |

| **Item** | | **Part 2. Coping** | | **Score** | | | | | | |  |  |
| --- | --- | --- | --- | --- | --- | --- | --- | --- | --- | --- | --- | --- |
| **9** | | You have difficulty proceeding through a congested intersection or rotary. | ① | | | ② | | ③ | | |  |  |
| **10** | | You have difficulty noticing moving people, signs, billboards, etc. while driving. | ① | | | ② | | ③ | | |  |  |
| **11** | | Because of your negligence while driving, traffic accidents occur frequently. | | | ① | | | ② | | ③ | | |
| **12** | | Passengers (e.g., children, family members, friends) are concerned about your ability to drive safely. | | | ① | | | ② | | ③ | | |
| **13** | | You have difficulty on or entering a fast-moving road, such as the highway. | | | ① | | | ② | | ③ | | |
| **14** | | You have difficulty in quickly responding to dangerous situations while driving. | | | ① | | | ② | | ③ | | |
| **15** | | Other drivers react negatively to your driving (e.g. flashing light, blowing horn). | | | ① | | | ② | | ③ | | |
| **16** | | Often surprised by a vehicle that you didn’t notice until it was quite close to you. | | | ① | | | ② | | ③ | | |
| **17** | | You have difficulty maintaining a safe distance from the preceding vehicle (e.g. lane change, rotation, parking). | | | ① | | | ② | | ③ | | |
| **18** | | You have difficulty focusing because of light (e.g., sunlight, another vehicle light, dashboard light). | | | ① | | | ② | | ③ | | |
| **19** | | You find it difficult to judge the moving speed of the vehicle (e.g., speed of approach to the vehicle in front, speed without looking at the speedometer). | | | ① | | | ② | | ③ | | |
| **20** | | You have difficulty quickly operating the brake (e.g., verifying the location of foot, strongly pushing) while driving. | | | ① | | | ② | | ③ | | |
| **21** | | If you get lost while driving, you have difficulty finding a route using the navigation or map. | | | ① | | | ② | | ③ | | |
| **22** | | You have difficulty driving and understanding the changing traffic rules. | | | ① | | | ② | | ③ | | |
| **23** | | You often hesitate at intersections without stop signs or red lights. | | | ① | | | ② | | ③ | | |
| **24** | | You have difficulty talking with passengers while driving. | | | ① | | | ② | | ③ | | |
|  | | | | | | | | | | | | |
| **Subtotal** | | | |  | | **/ 48 points** | | | | |  |  |

| **Item** | **Part 3. Health** | **Score** | | | |  |
| --- | --- | --- | --- | --- | --- | --- |
| **25** | You often forget the location of documents related to driving (vehicle registration, insurance policy), driver's license and keys. | ① | | ② | ③ |  |
| **26** | You have difficulty understand traffic guidance on the road. | ① | | ② | ③ |  |
| **27** | You often forget something pre-defined (go to the mart or gas station). | ① | | ② | ③ |  |
| **28** | You have difficulty finding your car in a parking lot | ① | | ② | ③ |  |
| **29** | You have difficulty finding a house by driving in familiar places (grocery stores, etc.). | ① | | ② | ③ |  |
| **30** | You often get tired while driving. | ① | | ② | ③ |  |
| **31** | You regularly verify your vision due to worry about its effect on the driving. | ① | | ② | ③ |  |
| **32** | You screw up your eyes to see distant objects and signs. | ① | | ② | ③ |  |
| **33** | You have difficulty reading the small print in the navigation or map. | ① | | ② | ③ |  |
| **34** | You have difficulty find a specific sign among many signs (e.g., finding a restaurant on the street) | ① | | ② | ③ |  |
| **35** | You have difficulty hearing a car horn, navigation voice guidance, etc. | ① | | ② | ③ |  |
| **36** | You experience pain or stiffness or weakness (in the hips, knees, ankles, and feet) during or after the driving. | ① | | ② | ③ |  |
| **37** | You feel decreased mobility (e.g., 2 m walk and climbing two floors without help) and flexibility (e.g., head turning while driving). | ① | | ② | ③ |  |
| **38** | You usually take medications (e.g., for sleep, blood pressure, depression, anxiety). | ① | | ② | ③ |  |
| **39** | You have consulted a doctor or pharmacist due to worry about the effect on driving of a medical condition. | ① | | ② | ③ |  |
| **40** | In the past 2 years, you have experienced dizziness, seizures, or loss of consciousness. | ① | | ② | ③ |  |
|  | | | | | |  |
| **Subtotal** | |  | **/ 48 points** | | | |
